# Effects of transcutaneous electrical nerve stimulation in the Management of Post-Injection Sciatic Pain in a non-randomized controlled clinical trial in Nnewi, Nigeria

**DOI:** 10.1186/s12906-018-2373-8

**Published:** 2018-11-26

**Authors:** Uchenna Prosper Okonkwo, Sam Chidi Ibeneme, Ebere Yvonne Ihegihu, Afamefuna Victor Egwuonwu, Ikechukwu Charles Ezema, Adesina Fatai Maruf, Emmanuel Chiebuka Okoye, Olanrewaju Peter Ibikunle, Antoninus Obinna Ezekwu

**Affiliations:** 1 Department of Physiotherapy, Nnamdi Azikiwe University Teaching Hospital, Nnewi, Anambra State PMB 5025 Nigeria; 2 Department of Medical Rehabilitation, Faculty of Health Sciences and Technology, University of Nigeria|, Enugu Campus, Enugu State Nigeria; 3 Department of Medical Rehabilitation, Faculty of Health Sciences and Technology, Nnamdi Azikiwe University, Awka, Anambra State Nigeria

**Keywords:** Sciatic nerve, Post-injection sciatic pain, Transcutaneous electrical nerve stimulation, Sham TENS

## Abstract

**Background:**

Many studies on transcutaneous electrical nerve stimulation (TENS) had been undertaken to explore its pain relieving efficiency on several medicals/surgical conditions but none, specifically, had been carried out to determine the effect it has on post-injection sciatic pain (PISP) which comes about from wrong administration of intramuscular pain. This study aims to assess the effects of TENS in the management of PISP.

**Methods:**

A total of 72 PISP subjects comprising 40 test subjects and 32 control subjects participated in a non-randomized controlled clinical trial in the current study. Participants were recruited from Department of Physiotherapy, Nnamdi Azikiwe University Teaching Hospital, Nnewi and Landmark Physiotherapy Services, Nnewi. The participants were however blinded to the intervention method they will receive before being allotted conveniently to test/experimental group (TG) or control group (CG). A written informed consent was obtained from participants before enrollments in the study. TENS and sham TENS (STENS) was applied to 40 test and 32 subjects respectively, 3 times a week, and 1 hour per session for the 10 weeks the study lasted. The Visual Analogue Scale was used to collect baseline data as well as those of 2nd, 4th, 6th, 8th and 10th weeks after TENS and STENS interventions. The data analysis was performed with the Descriptive statistic of Mean ± SD, mean comparison test, repeated analysis of variance and paired wise t-test. Statistical level of significance was set at *P* < 0.05.

**Result:**

Results of repeated measure ANOVA showed that the pain level among participants in the treatment group at the end (after 10 weeks) of the intervention was significantly lower than that of their counterparts in the control group (F = 16.26; *p* = 0.01); with the intervention accounting for the 19% of the variance. The effect size (partial eta squared) = 0.19.

**Conclusion:**

The outcome of this research has proved the effectiveness of TENS in the management of PISP and is being recommended in the management of PISP.

**Trial registration:**

Pan Africa Clinical Trial Registry (PACTR201805003408271). The study was registered retrospectively on the 29th May, 2018.

## Background

Nerve injection injury (NII) is a common complication following intramuscular injection; the sciatic nerve is the most frequently affected nerve [1, 2]. Sciatic nerves injection injury (SNII) has been recognized for many years: ‘sciatic neuritis due to injection’ was first reported in 1882 [3], sciatic nerve injuries were reported after quinine injections as early as 1920 [4]. However, SNII remains a persistent global problem that affects patients in both wealthy and poorer healthcare systems [5]. The World Health Organization has estimated that of the 12 billion injections administered globally every year, 50% of them are unsafely administered and 75% are unnecessarily administered [6]. Post-injection sciatic pain is a particular type of pain that stems from an injury to the sciatic nerve and its clinical presentations mimic that of sciatica only that its pain routes from the injection site downward. Due to its sensitive anatomical location and its supply of most of the muscles of the lower limbs the sciatic nerve is often times directly or indirectly traumatized during the process of administering an intramuscular injection or direct pressure on scar formation. The sciatic nerve can also be irritated by some other medical problems such as a herniating disc. The consequence of these on the body system is the generation of painful sensation that traverses partially or completely the route of the sciatic nerve and is known as sciatica. PISP has an intriguing nature and could present with the symptoms of pain, weakness, numbness and other discomforts along the sciatic nerve. It can afflict adults and non-adult from time to time and subsequently continues to interfere with the activities of daily living (ADL). There are varied causes/manifestations of pain; as such different medical options aimed at alleviating it may include surgical and non-surgical methods. The results of surgical approach or intervention in most cases are very disappointing. The non-surgical management involves administration of medicines, acupuncture, chiropractic, and physical therapy. One of the physical therapy modalities used in this regard is transcutaneous electrical nerve stimulation (TENS).

Significantly, when giving gluteal injections, it is safe to use the upper outer quadrant. The choice of site for injection must be based on good clinical judgment, using the best evidence available and individualized client assessment. There is wide agreement on the literature that the ventrogluteal site is preferable [7]. Review of the literature on relevant injection procedure found that injury to the sciatic nerve was associated with the use of the dorsogluteal site for injection. Sciatic pain affects one side of the lower limb; presenting with dull, sharp, or accompanied by intermittent shocks of shooting pain beginning at the buttock, travelling downward into the back or side of the thigh and / or leg. Sciatic pain then extends over the knees and may be felt in the feet. Sometimes symptoms may also include tingling sensation, sitting and trying to stand up is painful and difficult. Coughing and sneezing can intensify the pain [8]. Some medical disorders that can cause sciatica include: herniating discs, degenerative diseases of the lumbosacral spine, lumbar spinal stenosis, spondylolisthesis, spinal tumors, infections and Intramuscular injection [9]. The management of PISP can pose great difficulty to physicians and other medical professionals, as it sometimes does not arise immediately after an intramuscular injection. Authors experience in many years of clinical practice shows many clients have even forgotten about the injection experience. The modalities available for pain relief in physiotherapy practice include but not limited to infra-red radiation, manipulative therapy, interferential therapy, and Transcutaneous Electrical Nerve Stimulations (TENS), amongst others. Most times these options are used in combination in order to achieve maximal benefit [10].

For years, clinicians have been using TENS in an attempt to manage pain. It has been widely used in the treatment of various types of pain. It has also been shown that TENS is highly effective alleviating pain and reducing analgesic use following cesarean section, orthopedic and thoracic operations as well as mixed surgical procedures [11]. TENS is defined by the American Physical Therapy Association as the application of the electrical stimulation to the skin for pain relief [12]. Usually, the frequency, intensity, and pulse duration of the stimulation can be varied [10]. Conventional TENS is the most common mode used clinically and applies high frequency (> 50 Hz) and low intensity (below motor contraction, sensory only) stimulation parameters. Another common mode of stimulation uses low frequency (< 50 Hz) and high intensity (motor contraction) stimulation parameters [13]. Furthermore, increasing stimulation intensity to produce a painful noxious response is usually given at low frequency, and is called acupuncture-like TENS and is the least common [13].

Pain is a subjective sensation and therefore difficult to quantify. It is, however, important to quantify it for several reasons; one of the most compelling reasons is that assigning a measurement of pain gives patients a sense of control over their condition and has positive effects on their cop abilities. Pain measurements also provide a means of assessing the efficacy of response to treatment and prognosis. The Visual Analogue Scale (VAS) is a well-studied method of measuring both acute and chronic pain; its usefulness has been validated by several investigators [14–16].

Individuals who have PISP are often driven to seek relief from conventional medical treatment, alternative therapies, to miracle centers. One of the long-term effects being that many of the patients with PISP that were later referred for physiotherapy are in chronic stages of the problem. This study, therefore, examines the possibility of the use of TENS to bridge this gap. There were no previous empirical studies on the effect of TENS in managing PISP. The nearest were several case reports and a small number of controlled trials which reported improvements in pain symptoms in people with peripheral neuropathy or nerve damage [17, 18]. However, these studies suffer deficits of poor design or reporting hence additional researches are needed before a firm conclusion can be drawn about effectiveness. Consequently, there was not enough reliable evidence to draw a firm conclusion of this area [19, 20]. This lack of precedence over this research had created the problem of readymade standard protocol for a research of this nature. However; the empirical studies on the effect of TENS in managing other medical /surgical pains would be strongly relied upon. The working hypothesis is that there will be no significant difference between the test group and the control after 10 weeks of TENS and STENS application.

## Methods

The current study was a non-randomized controlled clinical trial involving seventy-two subjects − 40 test and 32 control participants. Participants were recruited from patients referred to Department of Physiotherapy, Nnamdi Azikiwe University Teaching Hospital, Nnewi and Landmark Physiotherapy Services, Nnewi. The purposive sampling technique was applied; all the subjects were required to meet certain selection criteria before participation in the study**.** Participants were blinded to the intervention they would receive by the investigator; two plain sheet papers had inscriptions T or C and folded. As participants came they were asked to pick either of the papers. Those that pick T will go to the TG while those that pick C will go to the CG. Ethical approval was sought from Nnamdi Azikiwe University Teaching Hospital Ethics Committee (NAUTHEC), Nnewi. A written informed consent was obtained from the participants in the study. The Visual Analogue Scale (VAS) was presented and described to participating subjects who were instructed to describe their level of pain by signifying a number on the VAS scale; 10 cm is the highest level of pain and 0 cm shows no pain. The baseline VAS scores were recorded for all the participants; it will constitute the basis of comparison of subsequent VAS scores. By this procedure, the mean pre and post VAS scores were obtained for the TG and CG at 2nd, 4th, 6th, 8th, and 10th weeks. The data analysis was performed with student t-test and independent t-test. Statistical level of significance was set at *P* < 0.05. The current study adheres to CONSORT guidelines.

### Sample size

The sample size determination was based on the 17% prevalence of injection palsy yearly as reported by Fatunde and Famulusi in Nigeria [21] and [22].

$$ {\displaystyle \begin{array}{c}\mathrm{Sample}\kern0.17em \mathrm{size}\left(\mathrm{n}\right)=\frac{Z^2\mathrm{p}\left(1-\mathrm{p}\right)}{{\mathrm{d}}^2}\\ {}\mathrm{p}=\mathrm{prevalence}=17\%=0.17\\ {}\mathrm{Z}=\mathrm{Z}\ \mathrm{statistic}\ \mathrm{for}\ 95\%\mathrm{level}\ \mathrm{of}\ \mathrm{confidence}=1.96\\ {}\mathrm{d}=\mathrm{precision}=0.05\end{array}} $$

it is recommended by various authors that a precision of 5% is appropriate for prevalence rates between 10 and 90% [22–24].

$$ \mathrm{n}=\frac{1.96^2\times 0.17\left(1-0.17\right)}{(0.05)^2} $$

$$ \mathrm{n}=\frac{3.8416\times 0.1411}{0.0025} $$

$$ \mathrm{n}=\frac{0.54197921}{0.0025} $$

$$ {\displaystyle \begin{array}{l}\mathrm{n}=217\\ {}\mathrm{Calculated}\ \mathrm{sample}\ \mathrm{size}=217\end{array}} $$

### Inclusion criteria

- the age range of 20 to 50 years
- post injection sciatic pain of not more than 1 year
- participants that stopped the medication for 2 weeks before intervention
- participants without foot drop
- participants without significant wasting of the muscles

### Exclusion criteria

- Spondylosis
- osteoarthritis of the knee
- metallic implant
- mentally unbalanced participants
- participants that refused to stop the medication
- very elderly people

## Intervention procedures

### Test group

Only TENS application was used on the 42 subjects that participated in the test group. Each patient was then made to lie on the available treatment plinth in a position (prone lying) that was comfortable and suitable for TENS application.

### The Electrodes Placement

**Fig. 1:**
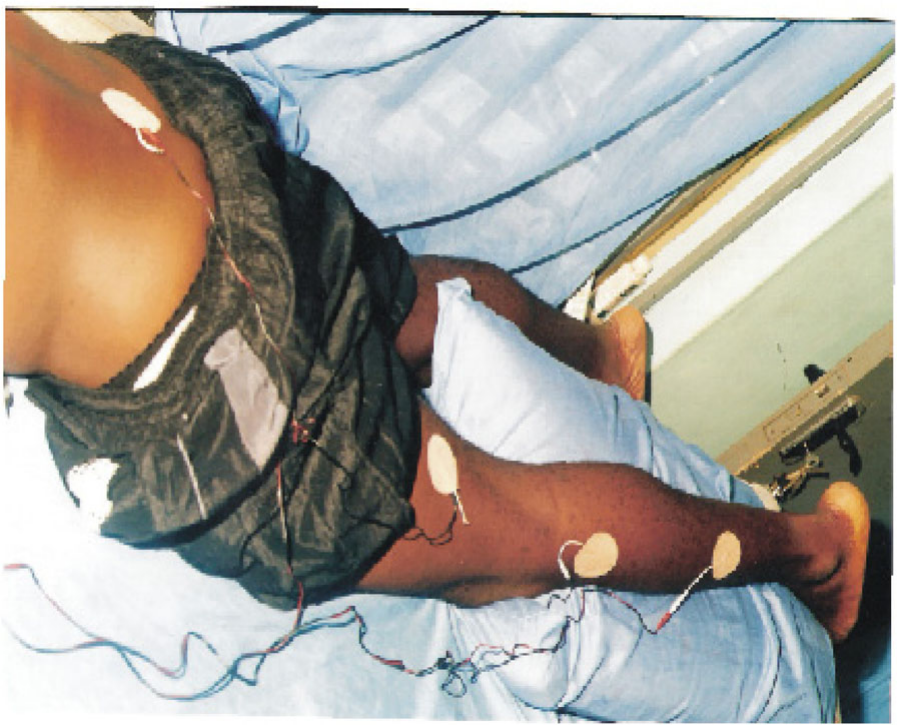
Showing the first tens electrodes placement on one of the subjects. The electrode was placed in such a way to cover the area of pain perception. From the sciatic nerve root origin down the route of sciatic nerve during a treatment session, in this position treatment lasted 20 min and electrodes changed to reflect the position in Fig. 2

**Fig. 2:**
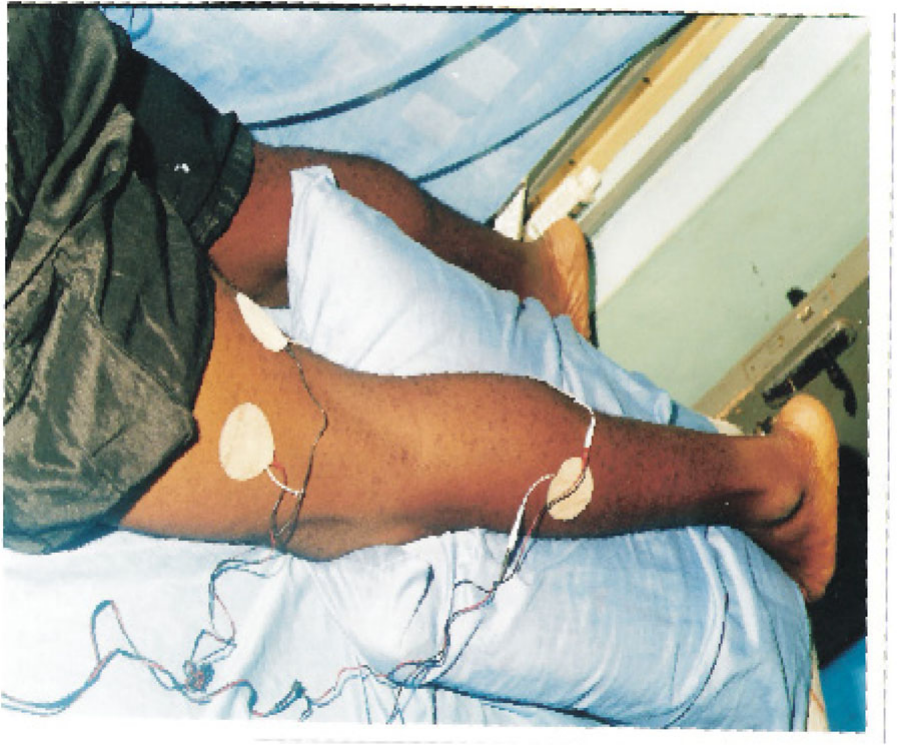
Showing the second tens electrode placement of on the same subject. The electrode was placed in such a way to cover the area of pain perception. Treatment lasted for 20 min and electrode placement was changed to reflect what is obtained in Fig. 3

**Fig. 3:**
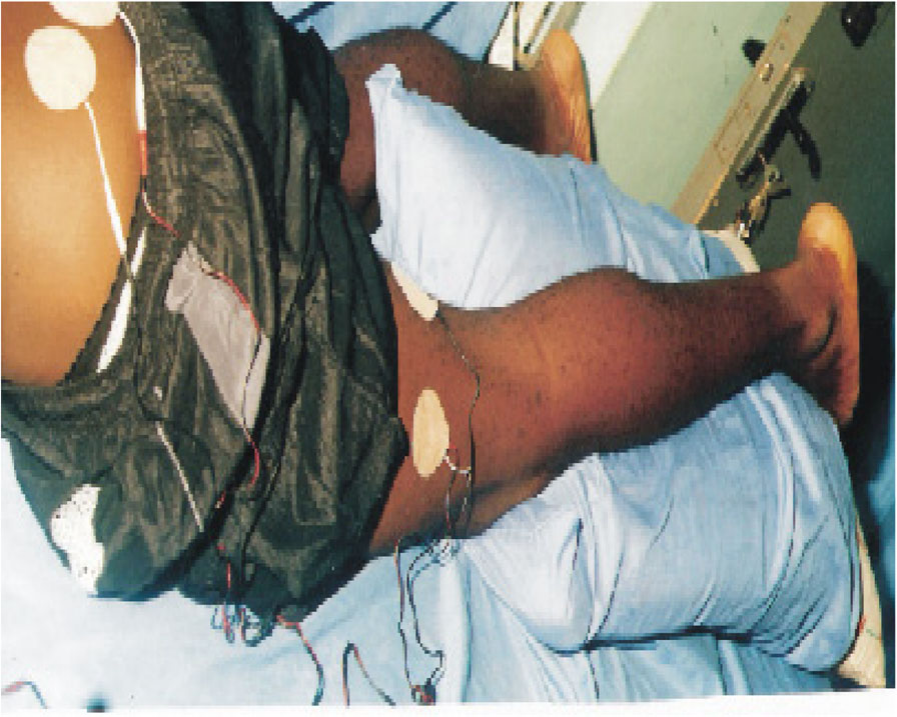
Showing the third tens electrode placement. This positioning also lasted 20 min. By this, the total treatment per session was 60 min for each subject

The adhesive electrodes were four in number in the dual channel type of TENS. They were securely placed along the route or course of the presenting sciatic pain (Figs. 1, 2 and 3) as maximum pain relief is obtained when the electrodes are placed on the painful area [25]. These electrode placement methods were applied throughout the period of study for the two groups. Patients’ education on the workings of TENS and skin toileting preceded the electrode application.

### TENS Mode

TENS which uses lower frequency stimulation (2-5 Hz) and a wider (longer) pulse width of (200-250*u*s) with an intensity greater than that of the traditional TENS was chosen because of its “carryover effect”. With all settings on zero, the TENS machine was switched on and the output increased until the patient perceives a fairly strong buzzing or pulsating sensation. The parameters of pulse frequency, pulse width, and pulse amplitude were varied from minimum to maximum rates of the chosen TENS mode to demonstrate the range to the subjects. The rate was varied (because each patient/subject felt and experienced each of these parameters differently) until the level that was most comfortable to the subject and which did not produce motor contraction was found. When the subject ceased to feel the stimulus after a few minutes probably because of nervous accommodation the output intensity was turned up until some strong sensation was felt again.

### Duration of TENS Application

Each subject received a total of 1 h of TENS per treatment session comprising 20 min per each 3 electrode placement methods. This added up to 3 h of TENS treatments per week which amounted to 30 h for the 10 weeks the study lasted.

### Control group (sham TENS)

The 32 subjects that fell into this group were available as a control group. The sham TENS application followed the same procedure of application except that the sham TENS was not switched on throughout the period the treatment lasted. To ensure that the participants were not biased they were not told the TENS modality on them were just a mere sham. The VAS scores before and after the application of the sham TENS were taking from the patients and appropriately recorded. The subjects positioning and other procedural formalities were the same as described for the test group above.

### Materials/equipment of study

- A dual channel TENS (EZ 105 Model) with a variable pulse frequency of 2-250 Hz, the variable pulse width of 50–250 microseconds and variable pulse intensity (amplitude) of 0-80 mA produced by Avionix Medical Devices, Texas, USA (Appendix 4) was used for both the test group and the control group. But that of the control was not activated to deliver electrical impulses to the control subjects.
- Stethoscope - Littman’s model
- Mercury Sphygmomanometer (Accusson model)
- Cotton and pin for skin sensation test.
- Measuring tape for muscle bulk measurement.
- Toilet soap, distilled water, and hand towel for skin cleansing.
- Visual analog scale by Price et al. [26]. Used for pain assessment pre and post-treatment.
- A Seca model weighing scale calibrated in a kilogram.
- A Seca model stadiometer calibrated in centimeter and inches.

### Statistical analysis

The SPSS software package version 23 was applied for data analysis. Descriptive statistic of Mean ± SD and mean comparison test was used to analyze baseline characteristics of subjects. Repeated analysis of variance was (ANOVA) was used to compare mean VAS scores between the TG and CG. A paired t-test was used for pair-wise comparison of pain level in each group across 10 weeks the Statistical level of significance was set at *P* < 0.05.

## Results

In Table 1 baseline characteristics shows 72 patients that participated in the study 40 were males while 32 were females. In the test group 40 patients participated, 24 were males while 16 were females. Their mean age was 29.737 ± 15.225 years and the mean duration of symptom was 3.95 ± 1.724. The mean body weight, height, and VAS were 55.700 ± 17.635 kg, 1.410 ± .1464 m and 6.23 ± 2.731 respectively. For the control group, of the 32 participants, 16 were males while 16 were females. The mean age for the control group was 38.409 ± 18.157 years and mean duration of symptom was 5.125 ± 1.738. The mean body weight, height, and VAS were 59.156 ± 8.648 kg, 1.41 ± .124 m and VAS 6.63 ± 2.297 respectively.

**Table 1:** Baseline characteristics of the groups evaluated at initial assessment

Variable	Experimental (*N* = 40)	Control (*N* = 32)
	Male = 24	Male = 16
	Female = 16	Female = 16
	Mean ± SD	Mean ± SD
Age (years)	29.737 ± 15.225	38.409 ± 18.157
Duration of symptom (months)	3.95 ± 1.724	5.125 ± 1.738
Weight (Kilogram)	55.700 ± 17.635	59.156 ± 8.648
Height (meters)	1.410 ± .1464	1.41 ± .124
VAS baseline	6.23 ± 2.731	6.63 ± 2.297

In Table 2 there was no significant difference between the test and control group for weight, height, and VAS at baseline. In contrast, there was a significant difference between test and control groups for age and duration of the symptom.

**Table 2:** Baseline mean comparison of age, duration of symptom, weight, height and visual analogue scale

Variable	Experimental	Control	*t* – value	*P* – value
	Mean ± SD	Mean ± SD		
Age (years)	29.737 ± 15.225	38.409 ± 18.157	−2.222	0.029*
Duration of symptom (months)	3.95 ± 1.724	5.125 ± 1.738	- 2.864	.006*
Weight (Kilogram)	55.700 ± 17.635	59.156 ± 8.648	- 1.014	.314
Height (meters)	1.410 ± .1464	1.41 ± .124	.-077	.939
VAS baseline	6.23 ± 2.731	6.63 ± 2.297	−.662	. 510

*significant at *p* < 0.05

Table 3 shows the mean pain levels (visual analog scale scores) of the participants in both experimental and control groups across 10 weeks. Unlike in the control group, there was a continuous decrease in pain levels in the experimental group across the duration of the study. Results of repeated measure ANOVA showed that the pain level among participants in the treatment group at the end (after 10 weeks) of the intervention was significantly lower than that of their counterparts in the control group (F = 16.26; *p* = 0.01); with the intervention accounting for the 19% of the variance (Table 4).

**Table 3:** Mean visual analogue scale scores of the participants in each group across 10 weeks

Time	Mean ± Standard deviation	
	Treatment	Control
Baseline Scores	6.23 ± 2.73	6.63 ± 2.30
After 2 Weeks	4.50 ± 3.07	6.09 ± 2.28
After 4 Weeks	3.63 ± 3.44	6.25 ± 2.17
After 6 Weeks	2.93 ± 3.15	6.28 ± 2.45
After 8 Weeks	2.73 ± 3.28	5.94 ± 2.03
After 10 Weeks	2.50 ± 3.23	5.50 ± 2.30

**Table 4:** Repeated measure ANOVA comparing the mean visual analogue scale scores between experimental and control groups after the intervention

Degree of freedom	Mean square	F	P	Partial Eta Squared
1	596.40	16.26	< 0.01*	0.19

*significant at *p* < 0.05

The paired comparison revealed that each of the pain level scores at the end of second, fourth, sixth, eightieth and tenth weeks was significantly lower than the baseline pain level score among the participants in the experimental group. In the experimental group, there was a significant difference in the pain levels in each pair of baseline, second, fourth, sixth, eightieth and tenth week (*p* < 0.05) except between pain levels at sixth and eightieth, and between those at eightieth and tenth weeks. In the control group, the baseline pain level was significantly lower than that at second, eightieth and tenth weeks (p < 0.05) (Table 5).

**Table 5:** Paired t test showing pair-wise comparison of pain level in each group across 10 weeks

(I) Time	Time (J)	T	P
Experimental Group			
Baseline	After 2 weeks	4.72	< 0.01*
	After 4 weeks	5.89	< 0.01*
	After 6 weeks	6.48	< 0.01*
	After 8 weeks	6.58	< 0.01*
	After 10 weeks	7.16	< 0.01*
After 2 weeks	After 4 weeks	2.49	0.02*
	After 6 weeks	4.26	< 0.01*
	After 8 weeks	4.43	< 0.01*
	After 10 weeks	5.19	< 0.01*
After 4 weeks	After 6 weeks	2.46	0.02*
	After 8 weeks	2.93	0.01*
	After 10 weeks	3.78	0.01*
After 6 weeks	After 8 weeks	1.48	0.15
	After 10 weeks	2.38	0.02*
After 8 weeks	After 10 weeks	1.22	0.23
Control Group			
Baseline	After 2 weeks	2.52	0.02
	After 4 weeks	1.34	0.19
	After 6 weeks	1.09	0.29
	After 8 weeks	2.87	0.01
	After 10 weeks	4.03	< 0.01
After 2 weeks	After 4 weeks	−0.50	0.62
	After 6 weeks	−0.63	0.54
	After 8 weeks	0.63	0.53
	After 10 weeks	1.93	0.06
After 4 weeks	After 6 weeks	−0.12	0.90
	After 8 weeks	1.26	0.22
	After 10 weeks	3.28	< 0.01
After 6 weeks	After 8 weeks	1.46	0.16
	After 10 weeks	2.91	0.01
After 8 weeks	After 10 weeks	1.60	0.12

*significant at *p* < 0.05

## Discussions

This study evaluated the effect of Transcutaneous Electrical Nerve Stimulation (TENS) in the management of sciatic pain following intramuscular injection. In carrying out the current study the authors noted that no similar studies had been done in the past regarding the effect of TENS in the management of PISP; however, the effect of TENS in managements other medical and surgical conditions were well documented. Interestingly, these studies used TENS device as adjunctive therapy, but most of the outcomes had not equivocally established combination therapy as producing lasting pain relief on the patients, This notwithstanding, the use of TENS in combination with other therapies were suggested by most previous studies in contrast to using it in monotherapy form as applied in this current study [18, 27]. The authors have noted that previous studies provided promising preliminary evidence about TENS but did not include clear descriptions of research design or results. This lack of detailed design has led to most of the published studies on TENS producing conflicting outcomes about the actual efficacy of TENS application. The authors had identified several factors which could contribute to these conflicting reports such as unspecified stimulation parameters, stimulation variables not controlled during the research process, different outcome measures, different electrodes placements, lack of placebo control, patients presenting at different stages in disease process, and failure to monitor or document patient’s compliance [18, 28–31]. The outcome of this current study has brought to limelight the importance of one of the physical therapy modalities in managing PISP.

**Fig. 4:**
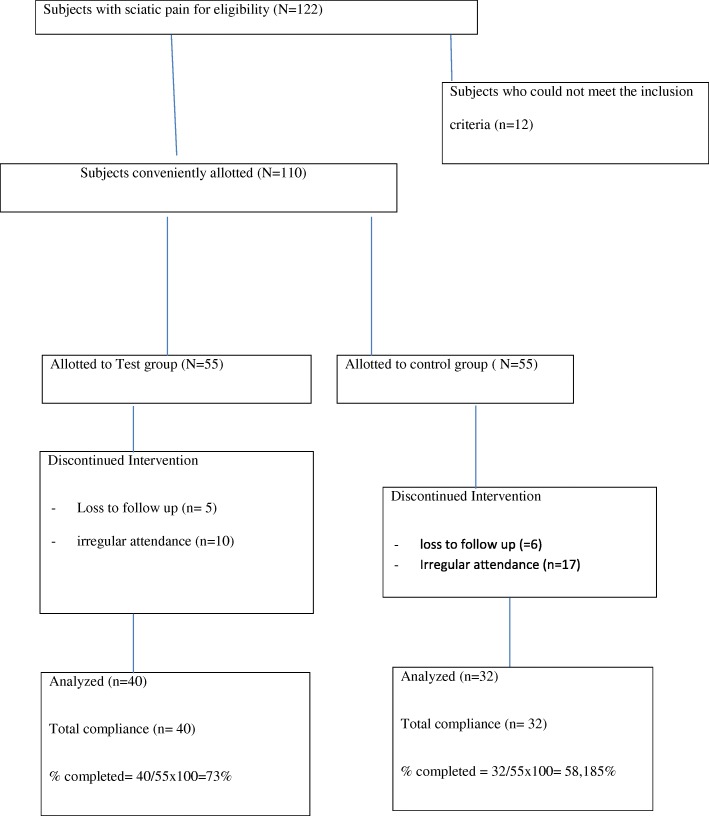
Pictorial comparison of the mean visual analog scale scores between the experimental and control groups at baseline, second, fourth, sixth, eightieth and tenth weeks. It shows the mean pain levels (visual analog scale scores) of the participants in both test/experimental and control groups across 10 weeks. Unlike in the control group, there was a continuous decrease in pain levels in the experimental group across the duration of the study. The baseline mean pain level for the test group was 6.23 ± 2.73 and after 10 weeks it decreased to 2.50 ± 3.23. The baseline mean pain level for the control group was 6.63 ± 2.30 and after 10 weeks it decreased to 5.50 ± 2.30

The result in Table 3 shows a trend in mean value variations between the two groups, unlike in the control group, there was a continuous decrease in pain levels in the experimental group across the duration of the study. Figure 4 shows the pictorial comparison of the mean pain levels between the two groups at baseline, second, fourth, sixth, eightieth and tenth weeks. This is an indication that TENS, a non-invasive modality, commonly used in physiotherapy is able to reduce PISP in the treatment group, unlike the placebo group. This study agreed with previous studies on the efficacy of TENS in pain management [32–34]. Specifically, the study by White et al. showed that TENS effectively decreased pains in 64 adults with disc herniation related sciatic pain by 23%, while the oral drugs intake was reduced by 15% [35]. This current study was done on the assumption that since TENS had been widely reported to be useful in managing various kinds of pain from dental procedures; osteoarthritis of the knee; angina pectoris; low back pain and chronic pain of all sorts; peripheral neuropathy to rheumatoid arthritis, that it could also be beneficial in managing PISP [17, 18, 36–42].

Results of repeated measure ANOVA showed that the pain level among participants in the treatment group at the end (after 10 weeks) of the intervention was significantly lower than that of their counterparts in the control group (F = 16.26; *p* = 0.01); with the intervention accounting for the 19% of the variance. The effect size (partial eta squared) was 0.19 (large) (Table 4). The clinical implication is that those in the test group responded better to TENS application than those who received STENS. Though there was some improvement in the control group as shown in Table 5 (pair-wise comparison of pain level), the authors were of the opinion that the said improvement, which might have resulted from the placebo effect and the possibility of subjects taking pain-relieving drugs, did not equal the improvement in the test group. The finding from the current study has rejected the working null hypothesis that there will be no significant difference (*p* > 0.05) between the TG and the CG after 10 weeks of TENS and STENS applications.

It has to be emphasized that TENS achieves its pain relief: pain gate mechanism or the endogenous opioid system. The variations in stimulation parameter used to activate these two systems will be briefly considered. Pain relief by means of the pain gate mechanism involves activation (excitation) of the beta sensory fibers, and by so doing reduce the transmission of the noxious stimulus from the ‘c’ fibers, through the spinal cord and on to the higher center. The Aβ fibers respond better when stimulated at a relatively high rate (in the order of 90–30 Hz or pps) but it is difficult to find support for the concept that there is a single frequency that works best for every patient, this range appears to cover the majority of individuals. An alternative approach is to stimulate the Aβ fibers which respond preferentially to a mode stimulation, which will activate the opioid mechanisms, and provide pain relief by causing the release of an endogenous opiate (encephalin) in the spinal cord. A third possibility is to stimulate both nerve types at the same time by employing burst mode stimulation. In this instance, the higher frequency stimulation output (typically at about 100 Hz) is interrupted (or burst) at the rate of 2 --3 bursts per second. When the machine is ‘on’, it will deliver pulses of the 100 Hz rate, thereby activating the Aβ and the pain gate mechanism, but by virtue of the rate of the burst, each burst will produce excitation in the A-delta fibers, therefore stimulating the opioid mechanisms. For some patients, this is by far the most effective approach to pain relief, though as a sensation, numerous patients find it less acceptable than other forms of TENS [43].

As applied to current study, 60-min cumulative treatment time was applied on PISP patients per session of treatment as against the recommended 20 or 30 min by previous TENS related studies, especially where TENS was used as addictive therapy modality. Consequent upon this, in carrying out this study, the authors were aware of likely depreciating effect of TENS overtimes probably due to adaptation to particular treatment mode and took measures to vary the parameters to minimize or avert it as it can negatively impact its efficiency in pain relief. This agrees with the scientific finding that the benefit of TENS tends to fall with time [44–46]. Also, depreciation in value-effect of TENS might be due to the adaptation of the nervous system to regular repetitive stimuli [47, 48]. The clinical implication of this is when TENS is applied for a long time as in current study nerve accommodation takes place and may affect the general efficacy of TENS in pain relief. To overcome anticipated accommodation effect in the current study the authors during treatment of TG patients were swinging between continuous and burst TENS modes [49]. The nerve adaptation and accommodation accounted for why some period after TENS is switched on, the patient complained that he/she is not feeling the buzzing or pulsating sensation well enough. Johnson et al. reported that individual patients used a specific pulse frequency but consistently there was a significant variation in the pulse frequency used by different patients [50]. The authors were also mindful of selecting the time of TENS applications because the stimulating effect does not start immediately but needs some time before its cumulative effect would be felt, this is in line with the outcome of experimental studies reported by previous authors [51, 52].

In the current study, from Table 2, there was a significant difference (*p* < 0.05) between the test and control groups for age and duration of symptom at baseline, this difference in baseline the authors noted could possibly have influenced the outcome of the study but no literature to back it up. Table 2 revealed no significant difference (*p* > 0.05) in the subjects’ baseline mean values of height, weight, and VAS between the test and control groups, this the authors assumed did not influence the outcome of this study.

**Fig. 5:**
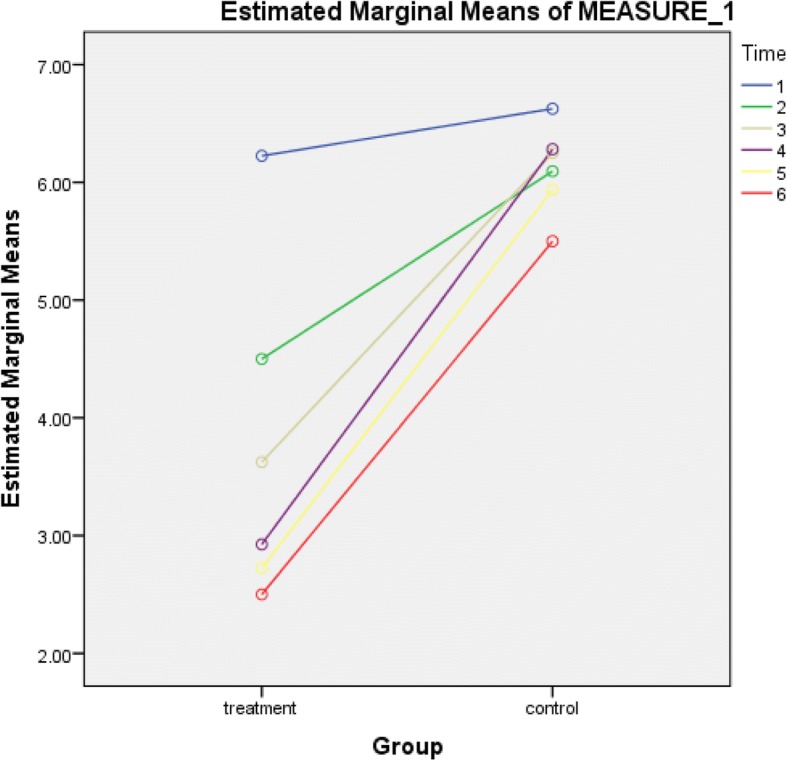
Subjects selection flow chart. Test group: 5 subjects absconded without any known reason, 10 subjects were irregular in attendance and the total that did not complete the study was 15. The total number that was eventually analyzed was 40. Control group: 6 subjects absconded without any known reason, 17 subjects were irregular attendance, and the total number that did not complete the study was 23 subjects. The total number that was eventually analyzed was 32 subjects

Moreover, deductions from the subjects’ recruitment flowchart (Fig. 5) showed more subjects in the CG either absconded or were irregular with treatment compared to what obtained in the TG. These differences might be attributed not only to a single factor but a variety of possible factors like not having the desired relief from pain, having good relief after few days of application of TENS, socio-economic and other considerations that are not within the immediate capacity of the authors to discern. Significantly, however, 40 of 55 subjects (73%) conveniently allotted to test group completed the study. Also, 32 of 55 subjects (58%) conveniently allotted to the control group completed the study.

The strength of the study lies in the use of VAS which is still generally accepted as a good tool for measuring variations in pain perception, the cost-effective nature of the measuring tool, the cooperation of the authors and the seemingly novel nature of the study which highlighted the application of TENS as a monotherapy treatment tool and one-hour application TENS for management of PISP.

The current study was however weakened by non-randomization of the samples, low sample size relative to the calculated sample size; the possibility that subjects in the groups still ingested one form of analgesic medication or the other; the subjective nature of pain assessment tool used, and the fact that the treatment modes of intensity, frequency and pulse width which varied amongst participants, and time did not provide for equal treatment of participants using a uniformed parameter**.** Furthermore, the significant differences in baseline VAS scores between age and duration of symptom could have influenced the outcome of the study.

## Conclusions

The outcome of the study showed significant improvement in PISP after 10 weeks TENS application. It also shows that STENS also achieved varied pain relief to control subjects but not significant enough to compare the effect of TENS on the test group. This has shown the usefulness of TENS in managing PISP of sub-acute and chronic nature. The implication for management and rehabilitation is that TENS alone is beneficial in the management of injection-related nerve pain as demonstrated from the outcome of the current study. A future line of study is consideration of comparative effects of TENS and TENS in combination in the management of PISP. Also, future studies that should factor the limitations highlighted above are advocated by the authors as it will help to strengthen quality, acceptability, and generalizability of the study outcome.

## Abbreviations

CG: control group
NAUTHEC: Nnamdi Azikiwe University Teaching Hospital Ethics Committee
NII: Nerve Injection Injury
PISP: post injection sciatic pain
SD: Standard division
SNII: Sciatic Nerve Injection Injury
STENS: sham transcutaneous electrical nerve stimulation
TENS: transcutaneous electrical nerve stimulation
TG: treatment group
VAS: Visual Analog scale
X: Mean
