# Correction to: Effects of transcutaneous electrical nerve stimulation in the Management of PostInjection Sciatic Pain in a non-randomized controlled clinical trial in Nnewi, Nigeria

**DOI:** 10.1186/s12906-018-2398-z

**Published:** 2019-01-08

**Authors:** Uchenna Prosper Okonkwo, Sam Chidi Ibeneme, Ebere Yvonne Ihegihu, Afamefuna Victor Egwuonwu, Ikechukwu Charles Ezema, Adesina Fatai Maruf, Emmanuel Chiebuka Okoye, Olanrewaju Peter Ibikunle, Antoninus Obinna Ezeukwu

**Affiliations:** 1 Department of Physiotherapy, Nnamdi Azikiwe University Teaching Hospital, Anambra State PMB 5025, Nnewi, Nigeria; 2 Department of Medical Rehabilitation, Faculty of Health Sciences and Technology, University of Nigeria|, Enugu Campus, Enugu State, Nigeria; 3 Department of Medical Rehabilitation, Faculty of Health Sciences and Technology, Nnamdi Azikiwe University, Awka, Anambra State Nigeria

## Correction to: BMC Complementary and Alternative Medicine (2018) 18:310 10.1186/s12906-018-2373-8

Following publication of the original article [1], the author reported that their family name was misspelled. The details are as follows:

**Incorrect name in the original article:**

 Antoninus Obinna Ezekwu.

**Correct name:**

 Antoninus Obinna Ezeukwu.

The original article has been corrected.
